# Effect of Tegoprazan on Tacrolimus and Mycophenolate Levels in Kidney Transplant Recipients: A Randomized Controlled Study Using a Smart Trial Platform

**DOI:** 10.3390/ph18060830

**Published:** 2025-06-01

**Authors:** Seong-Wook Lee, You Hyun Jeon, Jeong-Hoon Lim, Jung Ju Seo, Hee-Yeon Jung, Ji-Young Choi, Sun-Hee Park, Chan-Duck Kim, Yong-Lim Kim, Jang-Hee Cho

**Affiliations:** Division of Nephrology, Department of Internal Medicine, School of Medicine, Kyungpook National University Hospital, Kyungpook National University, Daegu 41944, Republic of Korea; maybee0914@gmail.com (S.-W.L.); yh-jeon@knu.ac.kr (Y.H.J.); jh-lim@knu.ac.kr (J.-H.L.); bravo2510@hanmail.net (J.J.S.); hy-jung@knu.ac.kr (H.-Y.J.); jyss1002@hanmail.net (J.-Y.C.); sh-park@knu.ac.kr (S.-H.P.); drcdkim@knu.ac.kr (C.-D.K.); ylkim@knu.ac.kr (Y.-L.K.)

**Keywords:** drug interactions, information and communication technology, kidney transplant, mycophenolate, potassium-competitive acid blocker, tacrolimus, tegoprazan

## Abstract

**Background/Objectives**: Potassium-competitive acid blockers (P-CABs) offer rapid gastric acid inhibition and lower toxicity compared to proton pump inhibitors (PPIs). This study investigates the drug–drug interaction between P-CABs and immunosuppressants tacrolimus and mycophenolate in kidney transplant recipients (KTRs). **Methods**: Sixty-two KTRs were randomized to receive either 50 mg of tegoprazan or 20 mg of pantoprazole. Patients were monitored using a smart clinical trial platform incorporating remote monitoring and safety management systems, which tracked drug adherence and vital signs. General and gastrointestinal (GI) symptoms were surveyed via a self-developed app on patients’ phones. Trough levels of tacrolimus and mycophenolate were measured every 4 weeks over 12 weeks. **Results**: Medication adherence was 100% in both groups. A total of 13,726 biometric data points and 5031 questionnaire responses were collected, with 5704 feedback messages and 56 video visits conducted. At 12 weeks, the mean trough levels of tacrolimus and mycophenolate were similar between the tegoprazan and pantoprazole groups (5.5 ± 1.6 vs. 5.8 ± 2.0 ng/mL, *p* = 0.50 and 2.7 ± 1.4 vs. 2.6 ± 1.4 µg/mL, *p* = 0.57, respectively). The intragroup difference in trough levels from baseline to week 12 was not significant in either group. GI symptoms scores, vital signs, and allograft function remained stable and comparable between groups. **Conclusions**: Tegoprazan does not alter the blood trough levels of tacrolimus and mycophenolate during the 12-week follow-up in KTRs and has a similar impact on GI symptoms as pantoprazole. This study confirms the feasibility and safety of using a smart clinical trial system with remote monitoring for randomized trials.

## 1. Introduction

Kidney transplantation represents the gold standard for treating end-stage renal disease, offering significant improvements in quality of life, patient survival, cost-effectiveness, and long-term outcomes compared to dialysis [1,2,3]. However, the primary challenge in kidney transplant recipients (KTRs) is rejection, which necessitates the continuous use of immunosuppressants to prevent graft failure [4]. The recommended immunosuppressive regimen for KTRs includes a triple combination therapy of tacrolimus, mycophenolate, and steroids [4,5,6].

Tacrolimus and mycophenolate are commonly associated with gastrointestinal (GI) side effects, such as dyspepsia, vomiting, and gastritis [6,7,8]. Proton pump inhibitors (PPIs) may be prescribed in KTRs to prevent or manage GI symptoms associated with immunosuppressive medication. Tacrolimus is metabolized by cytochrome P450 enzymes, and the concomitant use of PPIs can increase tacrolimus concentration, potentially leading to toxicity in patients with specific CYP2C19 and/or CYP3A5 genotypes through cytochrome or p-glycoprotein inhibition [9,10,11,12]. Additionally, several studies have demonstrated that PPIs reduce the absorption of mycophenolate mofetil [13,14], thereby decreasing exposure to its active compound, mycophenolic acid (MPA). Thus, therapeutic drug monitoring is crucial when PPIs are used, as they may affect the concentration of immunosuppressants.

Potassium-competitive acid blockers (P-CABs) are a newly developed class of gastric acid inhibitors that have gained widespread use for treating gastric diseases due to their rapid action and lower toxicity compared to traditional PPIs [15]. P-CABs achieve their therapeutic effect through direct binding to potassium-binding domains and do not require the activation of proton pumps [15,16]. Moreover, P-CABs reversibly bind to the proton pump and exhibit high stability in the acidic environment of the stomach, enabling prolonged inhibition and sustained effectiveness [15,16].

Tegoprazan, the first clinically available P-CAB, was approved in South Korea in 2018 for the treatment of erosive esophagitis and non-erosive reflux disease [16]. The pharmacokinetic and pharmacodynamic properties of tegoprazan are unaffected by food intake [17]. However, the potential drug–drug interactions between tegoprazan and tacrolimus, as well as mycophenolate, have not been investigated in KTRs.

In our previous randomized controlled trial, we evaluated immunosuppressive medication adherence using the smart clinical trial platform, comparing outcomes between an intervention group utilizing remote monitoring and a control group receiving standard care [18,19]. The operational feasibility demonstrated in that study provided the rationale for designing the present study as a randomized clinical trial comparing P-CABs and PPIs with the application of the information and communication technology (ICT)-based platform in a real-world setting. This study aimed to assess these potential drug interactions and evaluate the efficacy and safety of tegoprazan in KTRs using a smart clinical trial platform.

## 2. Results

### 2.1. Participants and Baseline Characteristics

**Figure 1:**
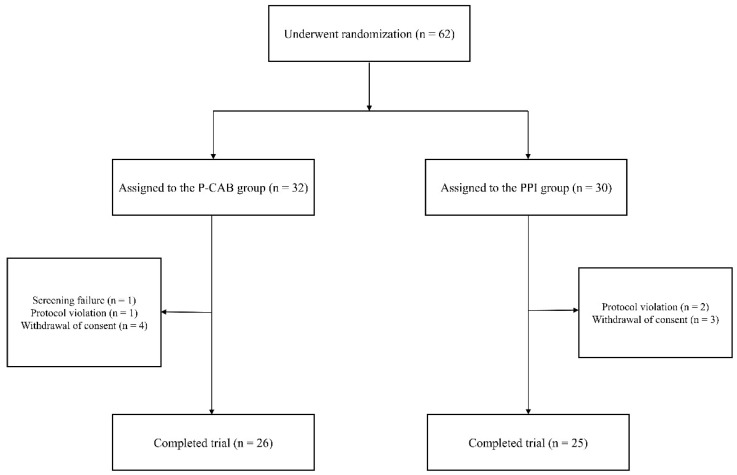
Flowchart of the study population. A total of 62 KTRs were randomly assigned to either the P-CAB group (tegoprazan 50 mg, n = 32) or the PPI group (pantoprazole 20 mg, n = 30). Following the exclusion of participants who either failed to adhere to the study protocol or withdrew their consent, 26 KTRs in the P-CAB group and 25 KTRs in the PPI group were included in the final analysis. Abbreviations: PPI, proton pump inhibitor; P-CAB, potassium-competitive acid blocker.

All enrolled KTRs (n = 62) were randomly assigned to either the P-CAB group (n = 32) or the PPI group (n = 30). Following the exclusion of participants who did not adhere to the study protocol or withdrew their consent, 51 KTRs completed the study, with 26 in the P-CAB group and 25 in the PPI group (Figure 1).

The baseline characteristics of the enrolled participants are detailed in Table 1. The mean age was 53.9 ± 10.6 years in the P-CAB group and 50.4 ± 8.3 years in the PPI group. Males constituted 69.2% of the P-CAB group and 68.0% of the PPI group. There were no significant differences between the groups in terms of baseline characteristics, including height, body mass index, serum creatinine levels, and eGFR.

### 2.2. Collected Data from the Smart Clinical Trial Platform

A total of 13,726 biometric data points were collected during the study period. The smart pill box recorded 4683 instances of medication data, with adherence to the study medication reaching 100% in both groups. The remote home monitoring system gathered 9043 biometric data points, including 8543 blood pressure readings, 265 body temperature recordings, and 235 heart rate measurements. Additionally, the electronic diary app received 5031 questionnaire responses, and the platform transmitted 5704 feedback messages. Non-face-to-face video visits were conducted for 56 regular and 5 unscheduled visits.

### 2.3. Effects of P-CABs and PPIs on Immunosuppressants

**Figure 2:**
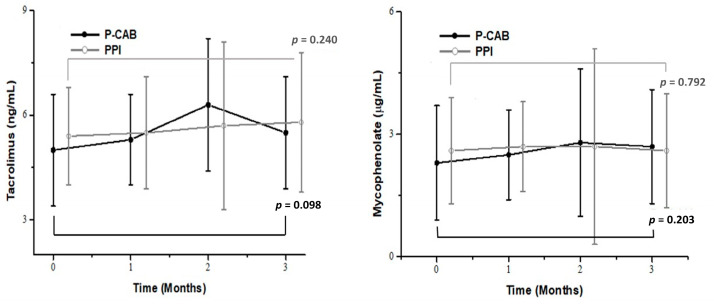
Comparison of trough levels of tacrolimus and mycophenolate in the P-CAB (black, closed circle) and PPI (gray, open circle) groups over a 12-week period. The intragroup differences in trough levels between baseline and week 12 were not significant in either group. Abbreviations: PPI, proton pump inhibitor; P-CAB, potassium-competitive acid blocker.

Table 2 presents the trough levels of tacrolimus and mycophenolate in the two groups. The baseline trough levels of tacrolimus and mycophenolate were similar between the groups (5.0 ± 1.6 ng/mL vs. 5.4 ± 1.4 ng/mL, *p* = 0.166 and 2.3 ± 1.4 µg/mL vs. 2.6 ± 1.3 µg/mL, *p* = 0.350, respectively). During the follow-up period, there were no significant differences in the tacrolimus trough levels between the groups at 4, 8, and 12 weeks (all *p* > 0.05). Similarly, no significant differences were observed in the mycophenolate trough levels at follow-up visits (all *p* > 0.05). The intragroup differences in trough levels between baseline and 12 weeks are illustrated in Figure 2, showing no significant changes in trough levels within either group (all *p* > 0.05).

**Figure 3:**
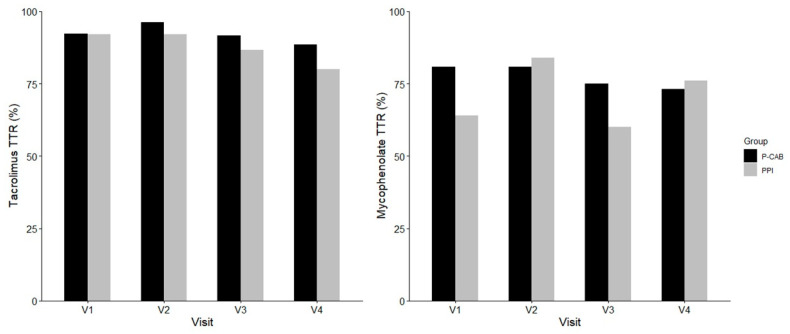
Comparison of time in therapeutic range (TTR) for tacrolimus and mycophenolate between P-CAB (black) and PPI (gray) groups across visits. TTR values were calculated at each scheduled visit. Abbreviations: PPI, proton pump inhibitor; P-CAB, potassium-competitive acid blocker; TTR, time in therapeutic range.

As shown in Figure 3, the time in therapeutic range (TTR) for tacrolimus and mycophenolate was comparable between the two groups across all visits. No significant differences were observed at any time point for either immunosuppressants (all *p* > 0.5).

### 2.4. Transplant Outcomes and GI Symptoms

The transplant outcomes are detailed in Table 3. Allograft function, assessed using eGFR, remained comparable at each visit across both groups (all *p* > 0.05). The incidence of de novo donor-specific antigen (DSA) did not differ significantly between the two groups. There were no occurrences of BPAR or allograft loss in any group during the study period.

Subjective GI symptoms were evaluated using the GERD-HRQL and RDQ, with the mean scores summarized in Table 4. Baseline GI symptom scores were similar between the two groups, and no significant differences were observed in the reduction in GI symptoms as measured by the questionnaires.

### 2.5. Adverse Events (AEs)

The overall incidence of AEs was 57.7% in the P-CAB group compared to 28.0% in the PPI group (*p* = 0.032). AEs were reported in 22 patients (43.1%), with the P-CAB group exhibiting a higher incidence of diarrhea and hypertension. Although two cases of serious adverse events (SAEs) were reported in the P-CAB group, none were related to the study drug. There was no significant difference in the incidence of individual AEs, SAEs, or early withdrawals due to AEs between the two groups (Table 5).

## 3. Discussion

This study enrolled 62 participants through appropriate random assignments, with 51 KTRs successfully completing the study using the smart clinical trial platform. Throughout the 12-week study period, none of the patients altered their dosage of immunosuppressants. Consequently, P-CAB administration did not result in statistically significant differences in the blood trough levels of tacrolimus and mycophenolate. Additionally, there were no significant differences observed in transplant outcomes, GI symptoms, and AEs during the study period.

Our smart clinical trial platform has been validated in a previous clinical trial [18,19]. In the trial, KTRs were divided into groups with and without remote monitoring using the smart clinical trial platform. The study did not demonstrate an improvement in adherence to immunosuppressants because of already high baseline adherence in this population. However, it provided a systemic basis for comparing intervention and standard care approaches, which supports the feasibility of comparative designs in this population. The present study was conducted to apply the system, whose feasibility had already been demonstrated, in the context of a randomized controlled trial evaluating drug-related outcomes.

In the present trial, an electronic diary app was developed to gather patient-reported outcomes more effectively, allowing patients to complete questionnaires at their convenience. The platform also incorporated non-face-to-face video visits, enabling regular video consultations and allowing medical staff to monitor patients’ conditions remotely. Patients were closely observed with remote home monitoring and received feedback messages. If a patient’s condition required medical staff attention, an unscheduled visit was conducted via the non-face-to-face video visit. Collectively, these enhancements suggest that our upgraded platform improved both the efficiency and safety of smart clinical trials.

The current study highlights not only the clinical implications of tegoprazan use in KTRs but also the operational potential of the smart clinical trial platform. This system integrates real-time data transmission, electronic drug monitoring, and remote patient assessments, enabling the accurate tracking of medication adherence and timely intervention. In this trial, individualized feedback was provided based on electronically recorded dosing behavior, resulting in a reported 100% adherence rate to the investigational drugs during the study period. While the present study focused on KTRs, the platform’s design and performance demonstrate its potential applicability to clinical trials across a variety of patient populations, especially in studies where precise adherence measurement and reinforcement are critical.

PPIs are extensively metabolized in the liver via cytochrome P450 enzymes, such as CYP2C19 and CYP3A4 [20]. Both PPIs and tacrolimus utilize the CYP3A4 enzyme for hepatic elimination, potentially leading to increased blood levels when used concurrently [20,21]. Several studies have reported that genetic variations in *CYP2C19*, *CYP3A5*, and *MDR1* may lead to increased blood levels of tacrolimus in KTRs taking PPIs [11,12]. Additionally, PPIs may alter the exposure of MPA by inhibiting gastric acid secretion [14,22].

However, several clinical studies have demonstrated that pantoprazole may be a safe option for patients receiving immunosuppressants [20]. A retrospective study involving 211 KTRs revealed no significant differences in tacrolimus trough levels and mycophenolate dose between the PPI group and the histamine-2 receptor antagonist group up to 12 months post-transplantation [23]. Lorf et al. investigated the blood levels of tacrolimus and cyclosporine in 12 solid organ transplant patients receiving pantoprazole, showing that five consecutive days of therapy with pantoprazole did not affect the trough levels of the immunosuppressants [24]. Bremer et al. also reported that the co-administration of pantoprazole does not influence the serum trough levels of tacrolimus in liver transplant recipients over a period of one year [25]. Additionally, a single-center study found that pantoprazole had no impact on the pharmacodynamics of MPA [26].

However, few studies have evaluated the drug interaction between P-CABs and immunosuppressants in KTRs. Two retrospective studies reported that switching from rabeprazole to vonoprazan increased the tacrolimus concentration in KTRs [27,28]. Conversely, Watari et al. demonstrated that conversion from rabeprazole to vonoprazan did not alter the tacrolimus trough levels regardless of CYP3A5 genotypes [29]. Our study distinguished itself by directly analyzing the impact of P-CABs compared to PPIs in a randomized controlled trial. This study is significant as it provides evidence that P-CAB use in KTRs did not significantly impact the blood concentrations of immunosuppressants over the study period. To our knowledge, no existing research has directly investigated the potential relationship between P-CAB intake and intrapatient variability in KTRs.

Some studies have reported that PPIs do not impact transplant outcomes, and our results align with these studies [30,31,32], showing no correlation between transplant outcomes and PPI administration. Additionally, our analysis demonstrated no meaningful changes in transplant outcomes between the two groups. Therefore, it appears that P-CAB administration might not exert a clinically significant effect on allograft kidney function in the short term.

Among the AEs, diarrhea and hypertension were reported only in the P-CAB group. However, patient interviews revealed that these symptoms were pre-existing conditions prior to enrollment, and no statistical significance was found between groups. Therefore, the causal relationship seemed low between P-CAB administration and the occurrence of diarrhea or hypertension in KTRs.

Our study demonstrated that P-CABs can safely replace PPIs for gastric prophylaxis in KTRs. However, several limitations exist. First, this study was conducted at a single center with a relatively small sample size, which may limit the generalizability of the findings. Second, the study focused exclusively on tegoprazan; therefore, the results may not be applicable to other P-CABs. To enhance the generalizability and clinical applicability of these findings, future multicenter trials with larger sample sizes and inclusion of diverse P-CAB agents are essential. Next, the follow-up duration of 12 weeks was relatively short and may not have been sufficient to detect delayed adverse effects. Longer-term studies are required to assess transplant outcomes such as changes in eGFR or the presence of BPAR and allograft loss. Lastly, we did not consider the association between P-CABs and cytochrome P450 gene polymorphisms, nor did we investigate the mechanisms behind the correlation of P-CABs with drug concentrations. Given that the pharmacokinetics of tacrolimus is sensitive to *CYP3A5* gene polymorphisms [21], further research on pharmacokinetics and pharmacogenetics is necessary to elucidate any potential relationships.

## 4. Materials and Methods

### 4.1. Study Design

This study was a prospective, open-label, randomized controlled trial conducted on KTRs receiving tacrolimus, mycophenolate, and steroids. This study was registered at the Clinical Research Information Service (CRIS), Republic of Korea (Registration No. KCT 0010481, registration date: 20 May 2025). Data analysis took place at Kyungpook National University Hospital, Republic of Korea, from August 2022 to May 2023. The trial adhered to the approval guidelines of the Institutional Review Board of Kyungpook National University Hospital (IRB No. 2022-02-013, approval date: 31 March 2022).

Inclusion criteria for the participants were as follows: age 19 years or older, a history of kidney transplantation only (without other organ transplants), use of tacrolimus, mycophenolate, and steroids for immunosuppression after kidney transplantation, stable tacrolimus trough levels maintained between 3 and 8 ng/mL, ability to provide handwritten informed consent, and capacity to attend scheduled clinical research visits. Participants also needed to have experienced heartburn symptoms for ≥1 month and to have received immunosuppressant treatment for ≥12 weeks.

Exclusion criteria comprised refusal of ICT-based centralized monitoring, history of treatment for acute rejection within the last 3 months, presence of active infectious disease, visual or hearing impairment, inability to operate limbs, and skin hypersensitivity reactions that could interfere with the use of smart pill boxes and blood pressure devices. Additionally, pregnancy, lactation, and non-compliance with appropriate contraceptive use during the trial were also criteria for exclusion.

We determined the sample size based on a study that compared MPA exposure after PPIs [22]. The mean difference was 18, and the estimated standard deviation was 20. Assuming 80% power and a two-sided type 1 error rate of 5%, the required number of subjects was 25 in each arm. We also predicted a 20% dropout rate for both groups, so the total sample size was calculated to be 62.

Patients were randomly assigned to either the P-CAB (tegoprazan 50 mg) group or the PPI (pantoprazole 20 mg) group. Enrolled KTRs were scheduled for four visits at baseline, 4, 8, and 12 weeks post-randomization. The dosage of immunosuppressants remained unchanged throughout the 12-week study period. Blood tests, including measurements of immunosuppressant trough levels, serum creatinine levels, estimated glomerular filtration rate (eGFR), and vital signs, were conducted at each visit.

### 4.2. Smart Clinical Trial Platform: Components and Collected Information

**Figure 4:**
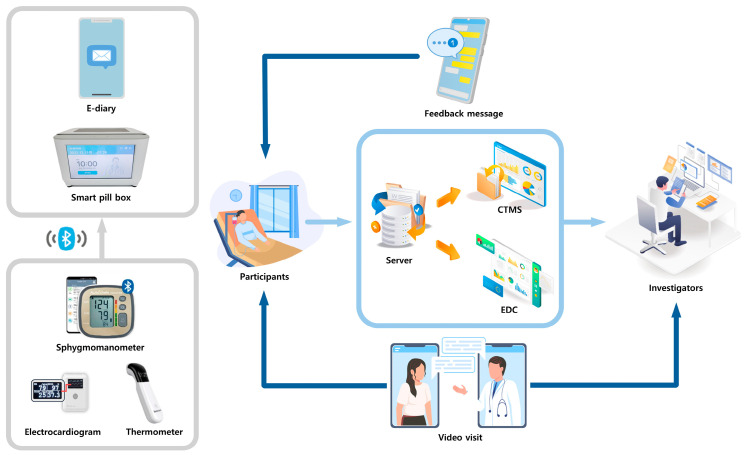
Schematic representation of the smart clinical trial system. The smart clinical trial platform integrates a smart pill box, remote home monitoring, an electronic case report form, a comprehensive clinical trial management system, an electronic diary application, non-face-to-face video consultations, and safety management systems.

The smart clinical trial platform comprises several integrated components, including a smart pill box, remote home monitoring systems, an electronic case report form (eCRF), a comprehensive clinical trial management system (CTMS), an electronic diary application, non-face-to-face video consultations, and safety management systems (Figure 4). The smart pill box and remote home monitoring system gather data on medication adherence, blood pressure, pulse rate, body temperature, and electrocardiograms. These data are transmitted to the system server, where it is compiled into the eCRF and CTMS. General and GI symptom questionnaires are administered via a self-developed electronic diary app installed on the patient’s phone. One non-face-to-face video visit is scheduled during the study period. Safety management systems are designed to send text alarms to both patients and investigators in the event of abnormal data. Through this monitoring system, participants and medical staff receive feedback via text messages and pillbox alarms for medication administration errors, incorrect timing, missed dosages, or other biological data discrepancies [19].

### 4.3. Smart Pill Box

Upon registration in the CTMS, participants receive education on the smart pill box and remote home monitoring system at hospitals. The smart pill box records information on medication use, doses ingested, doses remaining, and dosing times. It connects to remote home monitoring devices via Bluetooth, acting as a gateway to transmit collected data to the system server. This device ensures the accurate recording and transmission of medication intake information in a home environment.

### 4.4. Remote Home Monitoring System

The remote home monitoring system includes the smart pill box and various monitoring devices responsible for acquiring and transmitting patient data. In this trial, the system comprised a smart pill box, a sphygmomanometer for blood pressure and pulse, a thermometer for body temperature, and an electrocardiogram for heart rate. Medication adherence was monitored daily, blood pressure and pulse were measured five days a week, and body temperature and electrocardiogram data were recorded monthly.

### 4.5. Electronic Diary App

Changes in GI symptoms were measured and recorded using a self-developed electronic diary app installed on participants’ mobile phones. This app facilitated the assessment of GI symptoms through the Gastroesophageal Reflux Disease–Health-Related Quality of Life (GERD-HRQL) and Reflux Disease Questionnaire (RDQ). The GERD-HRQL scale comprises 10 items, addressing both GI symptoms and the patient’s overall condition. Each item is scored on a scale from 0 to 5, with higher scores indicating a poorer quality of life. The RDQ is a 6-item self-administered questionnaire designed to evaluate the severity and frequency of heartburn sensation, gastric acid regurgitation, and dyspepsia.

### 4.6. Non-Face-to-Face Video Visit

**Figure 5:**
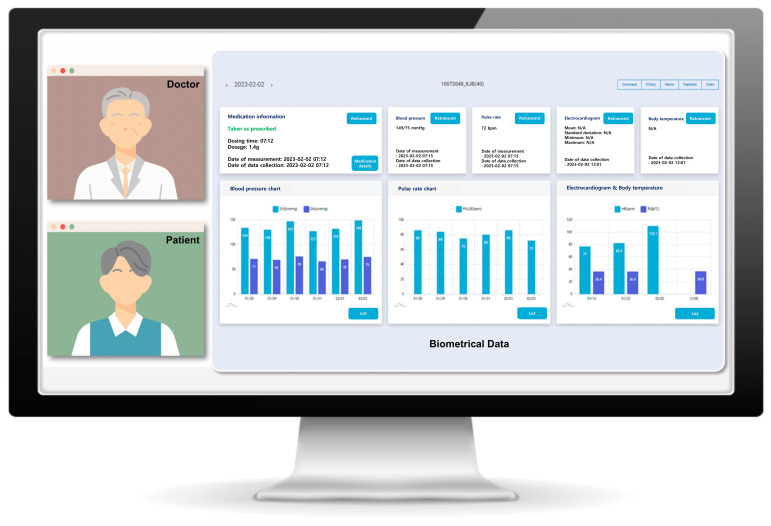
Interface of the non-face-to-face visit system. This system is linked to the electronic case report form, enabling simultaneous monitoring of medication adherence and biometric data.

This system enabled participants and medical staff to conduct non-face-to-face visits, eliminating the need for physical hospital visits. The interface of the non-face-to-face visit system was integrated with the eCRF, allowing for the simultaneous monitoring of medication adherence and biometrical data (Figure 5). Medical staff could perform visits akin to traditional hospital visits by asking questions and receiving responses from participants. Non-face-to-face video consultations were conducted on the third visit and could also replace unscheduled visits.

### 4.7. Safety Management System

Participants and medical staff received text message alarms regarding missed doses, misuse, and overuse of medications. In cases of abnormal biometrical data, predefined feedback messages were sent to participants and medical staff to ensure the safety of the clinical trials. If the abnormal event persisted, participants were instructed to make an unscheduled visit, which could be conducted via a non-face-to-face video consultation or an in-person hospital visit.

### 4.8. Data Collection

The collected data included patient demographics such as gender, age, height, weight, blood tacrolimus, mycophenolate concentrations, kidney function, liver function tests, allograft loss, and acute rejection episodes. We collected DSA information. DSA was identified using the Luminex single-antigen assay (LSA). These data were collected through a review of the eCRF and medical records.

### 4.9. Study Outcomes

The primary outcome was to compare changes in drug concentrations of tacrolimus and mycophenolate following the administration of tegoprazan in KTRs.

The secondary outcome included evaluating transplant outcomes and GI symptoms using questionnaires. The transplant outcomes assessed were changes in eGFR, biopsy-proven acute rejection (BPAR), allograft loss, and de novo DSA during the study period. De novo DSA was defined as the new appearance of antibodies against mismatched donor human leukocyte antigen (HLA) not present at baseline.

Safety evaluations were conducted through comprehensive monitoring, including physical examinations, clinical laboratory tests, vital signs, and the documentation of adverse events (AEs). Participants were encouraged to voluntarily report AEs regularly throughout the study.

### 4.10. Measurements of the Immunosuppressant Trough Level

Trough levels of tacrolimus and mycophenolate were measured at 12 h post-dose, following standard clinical practice for therapeutic drug monitoring in transplant recipients [33]. Blood samples were collected at scheduled morning visits, and the 12 h interval was confirmed using electronic pillbox adherence data or patient interviews. Tacrolimus concentrations were measured using the Dimension^®^ TAC Flex^®^ reagent cartridge (Siemens Healthcare Diagnostics Inc., Newark, NJ, USA) on the Siemens Dimension^®^ Integrated Chemistry System, employing a homogeneous chemiluminescent immunoassay (CLIA) method. Mycophenolic acid concentrations were measured using the Dimension^®^ MPA Flex^®^ reagent cartridge (Siemens Healthcare Diagnostics Inc., Newark, USA) on the same system, based on a particle-enhanced turbidimetric inhibition immunoassay (PETINIA) method. All assays were conducted in the Department of Laboratory Medicine at the study hospital.

### 4.11. Statistical Analysis

Continuous variables were expressed as mean ± standard deviation, while categorical variables were presented as numbers and percentages. For normally distributed data, *t*-tests were utilized; for non-normally distributed data, the Mann–Whitney U test was applied. Intragroup variations between baseline and 12 weeks were analyzed using paired *t*-tests. Differences in the frequency of transplant outcomes were assessed using the χ^2^ test or Fisher’s exact test. Differences in GI symptom questionnaire scores, assessed via e-diary surveys, were analyzed using *t*-tests or the Mann–Whitney U test, as appropriate. We conducted a TTR analysis for both tacrolimus and mycophenolate to evaluate the potential effects of tegoprazan on immunosuppressant stability. The predefined therapeutic range was set at 3–8 mg/mL for tacrolimus and at 1–3.5 µg/mL for mycophenolate. TTR was calculated as the proportion of time each drug level remained within the therapeutic window across the study period. All statistical analyses were performed using IBM SPSS Statistics 25 for Windows, version 22. A *p*-value of <0.05 was considered statistically significant.

## 5. Conclusions

In conclusion, this smart clinical trial demonstrated no significant differences in the blood trough levels of tacrolimus and mycophenolate between the two groups. P-CAB, with its rapid and robust suppression of gastric secretion, exhibited a comparable efficacy in alleviating GI symptoms in KTRs relative to PPIs. Although no significant differences in transplant outcomes were observed during the 12-week follow-up period, the smart clinical trial system, utilizing non-face-to-face video visits, proved to be both effective and safe in conducting randomized trials.

However, due to constraints related to sample size and duration of follow-up, the findings should be regarded as preliminary. It is essential to conduct larger and more extensive studies to validate the safety and pharmacological neutrality of P-CABs within the context of transplantation.

## Figures and Tables

**Table 1:** Baseline characteristics for the enrolled patients.

Variables	P-CAB (n = 26)	PPI (n = 25)	*p*-Value
Age (year)	53.9 ± 10.6	50.4 ± 8.3	0.204
Height (cm)	167.9 ± 7.1	165.5 ± 8.5	0.270
Body weight (kg)	60.1 ± 12.2	62.1 ± 12.2	0.127
BMI (kg/m^2^)	22.6 ± 3.2	22.5 ± 2.6	0.741
Gender (male)	18 (69.2)	17 (68.0)	0.925
BUN (mg/dL)	19.5 ± 6.3	20.0 ± 7.0	0.679
Creatinine (mg/dL)	1.1 ± 0.3	1.2 ± 0.4	0.178
Glucose (mg/dL)	115.7 ± 30.1	110.4 ± 22.6	0.940
eGFR (mL/min/1.73 m^2^)	62.8 ± 17.1	57.7 ± 14.9	0.397
AST (U/L)	17.6 ± 4.6	17.7 ± 4.3	0.934
ALT (U/L)	15.3 ± 6.1	13.9 ± 5.1	0.383
GTP (U/L)	32.3 ± 29.5	23.1 ± 19.4	0.117

Data are shown as mean ± standard deviation or number (%). Abbreviations: ALT, alanine aminotransferase; AST, aspartate aminotransferase; BMI, body mass index; BUN, blood urea nitrogen; eGFR, estimated glomerular filtration rate; GTP, gamma-glutamyl transferase; PPI, proton pump inhibitor; P-CAB, potassium-competitive acid blocker.

**Table 2:** Trough levels of the immunosuppressants.

Immunosuppressants		P-CAB (n = 26)	PPI (n = 25)	*p*-Value
Tacrolimus (ng/mL)	Baseline	5.0 ± 1.6	5.4 ± 1.4	0.166
	Week 4	5.3 ± 1.3	5.5 ± 1.6	0.598
	Week 8	6.3 ± 1.9	5.7 ± 2.4	0.479
	Week 12	5.5 ± 1.6	5.8 ± 2.0	0.500
Mycophenolate (µg/mL)	Baseline	2.3 ± 1.4	2.6 ± 1.3	0.350
	Week 4	2.5 ± 1.1	2.7 ± 1.1	0.515
	Week 8	2.8 ± 1.8	2.7 ± 2.4	0.294
	Week 12	2.7 ± 1.4	2.6 ± 1.4	0.565

Data are shown as mean ± standard deviation. Abbreviations: PPI, proton pump inhibitor; P-CAB, potassium-competitive acid blocker.

**Table 3:** Transplant outcomes of the patients.

	eGFR (mL/min/1.73 m^2^)				De Novo DSA	BPAR	Allograft Loss
	Baseline	Week 4	Week 8	Week 12			
P-CAB (n = 26)	62.8 ± 17.1	61.8 ± 13.0	60.6 ± 12.8	63.5 ± 16.5	1 (4.2%)	0	0
PPI (n = 25)	57.7 ± 14.9	60.1 ± 14.8	63.6 ± 14.1	60.6 ± 14.7	2 (8.3%)	0	0
*p*-value	0.397	0.538	0.569	0.618	1.000	N/A	N/A

Data are shown as mean ± standard deviation or number and number (%). Abbreviations: BPAR, biopsy-proven acute rejection; DSA, donor-specific antigen; eGFR, estimated glomerular filtration rate; N/A, not applicable; PPI, proton pump inhibitor; P-CAB, potassium-competitive acid blocker.

**Table 4:** Comparison of questionnaire scores for the gastrointestinal tract.

Questionnaire for Quality of Life		P-CAB (n = 26)	PPI (n = 25)	*p*-Value
GERD-HRQL	Baseline	4.9 ± 4.3	2.8 ± 2.3	0.074
	Week 4	0.9 ± 1.4	0.7 ± 1.2	0.400
	Week 8	0.7 ± 1.0	0.9 ± 1.8	0.583
	Week 12	0.7 ± 1.6	0.5 ± 1.1	0.382
RDQ	Baseline	4.7 ± 4.7	3.2 ± 3.3	0.426
	Week 4	1.0 ± 2.1	0.3 ± 0.9	0.322
	Week 8	0.6 ± 1.2	0.4 ± 1.0	0.617
	Week 12	0.7 ± 1.9	0.7 ± 2.0	0.822

Data are shown as mean ± standard deviation. Abbreviations: GERD-HRQL, Gastroesophageal Reflux Disease–Health-Related Quality of Life; RDQ, Reflux Disease Questionnaire; PPI, proton pump inhibitor; P-CAB, potassium-competitive acid blocker.

**Table 5:** Number of participants with adverse events.

	P-CAB (n = 26)	PPI (n = 25)	*p*-Value
AEs	15 (57.7)	7 (28.0)	0.032
SAEs	2 (7.7)	0 (0.0)	NA
Withdrawal due to AEs	0 (0.0)	0 (0.0)	NA
AEs			
Abdominal discomfort	3 (11.5)	2 (8.0)	1.000
Arthralgia	1 (3.8)	0 (0.0)	1.000
Constipation	2 (7.7)	0 (0.0)	0.490
COVID-19	0 (0.0)	2 (8.0)	0.235
Diarrhea	5 (19.2)	0 (0.0)	0.051
Headache	0 (0.0)	1 (4.0)	0.490
Hypertension	4 (15.4)	0 (0.0)	0.110
Hypoglycemia	1 (3.8)	0 (0.0)	1.000
Myalgia	0 (0.0)	1 (4.0)	0.490
Nausea, vomiting	3 (11.5)	0 (0.0)	0.235
Productive cough, sputum	4 (15.4)	0 (0.0)	0.110
Pyrexia	0 (0.0)	1 (4.0)	0.490
Toothache	0 (0.0)	1 (4.0)	0.490
Upper respiratory tract infection	2 (7.7)	1 (4.0)	1.000
Urinary tract infection	0 (0.0)	1 (4.0)	0.490
Vertigo	0 (0.0)	1 (4.0)	0.490

Data are shown as number (%). Abbreviations: AE, adverse event; SAE, serious adverse event; NA, not applicable; PPI, proton pump inhibitor; P-CAB, potassium-competitive acid blocker.

## Data Availability

The data presented in this study are available on request from the corresponding author. The data are not publicly available due to privacy or ethical restrictions.

## Abbreviations

The following abbreviations are used in this manuscript:

AEs	Adverse events
ALT	Alanine aminotransferase
AST	Aspartate aminotransferase
BPAR	Biopsy-proven acute rejection
BMI	Body mass index
BUN	Blood urea nitrogen
CTMS	Clinical trial management system
eCRF	Electronic case report form
eGFR	Estimated glomerular filtration rate
GERD-HRQL	Gastroesophageal Reflux Disease–Health-Related Quality of Life
GI	Gastrointestinal
GTP	Gamma-glutamyl transferase
KTRs	Kidney transplant recipients
MPA	Mycophenolic acid
P-CABs	Potassium-competitive acid blockers
PPIs	Proton pump inhibitors
RDQ	Reflux Disease Questionnaire
SAEs	Severe adverse events
